# Approaches to learning in pre-medicine: a multi-university mixed-methods study

**DOI:** 10.1186/s12909-025-08228-x

**Published:** 2025-11-19

**Authors:** Declan Gaynor, Fiza Rashid-Doubell, Celine J Marmion, Isabel Stabile

**Affiliations:** 1 School of Medicine, RCSI Bahrain, P.O. Box 15503, Adliya, Bahrain; 2 Faculty of Dental Surgery, University of Malta, Msida, Malta; 3 Department of Chemistry, RCSI University of Medicine and Health Sciences, 123 St. Stephen’s Green, Dublin 2, Ireland

**Keywords:** Approaches to learning, ASSIST, Academic performance, Mixed-methods, Medicine.

## Abstract

**Background:**

In the approaches to learning model, it is proposed that learners’ methodologies are acquired and adjusted according to the environment and context. Learners can be classified as adopting deep, surface or strategic approaches to learning. Understanding learners’ preferences and the factors associated with these approaches can benefit their learning journey.

**Methods:**

We conducted an exploratory multi-university mixed-methods cross-sectional study of pre-medicine foundation/pathway students at three medical schools (Europe/Middle East). Students completed the short-form ASSIST (Deep, Strategic, Surface); *n* = 159. We assessed internal consistency, compared ASSIST scale scores across groups using non-parametric tests, examined correlations between ASSIST scales and academic performance, and ran a hierarchical linear regression as a confirmatory test. We purposively sampled students (*n* = 25) based on learning approach and background, and completed semi-structured interviews. Data were analysed using thematic analysis.

**Results:**

Students most commonly reported Strategic (21%), Deep (21%), or Deep–Strategic (29%) preferences. Between-site differences were detected for Deep (Dublin < Bahrain; small) and Surface (Malta < Dublin and Bahrain; small–to–moderate), with no site effect for Strategic. No differences were observed by gender or academic achievement on entry. English proficiency (B2 > C2) and prior education (CHSD < prior foundation programme) showed differences on the Deep scale. The Strategic scale correlated positively with academic performance (*r* ≈ 0.23), but this attenuated and lost conventional significance in regression once entry achievement and background were included. Qualitative interviews revealed that students switch approaches based on subject, task, and assessment demands. Organisation, time-management, utilisation of resources, and assessment awareness were strong features in strategic approach learners, while surface learners reflected a passive, memorisation-focused approach under pressure.

**Conclusions:**

Across three international cohorts, Deep and Strategic approaches predominated, with modest between-site differences, and approach–achievement links were limited after accounting for background factors. Qualitative data highlighted context-responsive switching and the role of practical strategic behaviours in managing assessment demands. Rather than aiming to shift students to a single “ideal” approach through curriculum change, programmes may achieve more by teaching self-regulated study skills, refining assessment/feedback to discourage unhelpful surface tactics, and targeting transition supports for students with differing educational and language backgrounds.

**Supplementary Information:**

The online version contains supplementary material available at 10.1186/s12909-025-08228-x.

## Background

As health science educators, we are continuously searching for ways to improve the experience of our students and, in turn, the quality of our graduates, the next generation of health science professionals. One key aspect of a student’s educational experience is the nature of the learning approach that they employ during their education. A learning approach is defined as the way students relate to a learning task to achieve the resulting outcome. Much of the current research on approaches to learning is based around the work and refinements of Marton and Saljo [1], Biggs [2] and Entwistle and Ramsden [3], which identified three different approaches to learning: Deep, Surface Apathetic and Strategic. Several different instruments have been devised to describe and examine the concepts and constructs found in these approaches to learning by students in higher education. These include the Study Processes Questionnaire, and its revised version (SPQ and R-SPQ-2 F) [4], the Learning and Study Strategies Inventory (LASSI) [5], the Approaches to Study Inventory (ASI) [3], and an evolution of the ASI known as the Approaches and Study Skills Inventory for Students (ASSIST) [6].

It is proposed that approaches to learning are acquired rather than inherent and can be adjusted according to the environment and the context of learning [7]. The deep approach emphasises understanding and relating ideas, while the surface approach is syllabus-bound and superficial in nature, with a reliance on rote memorisation. The strategic approach is focused on maximising achievement in academic assessments, with considerable effort invested in time management, study organisation, and assessment strategies and less emphasis on understanding and relating concepts.

Recognition of a student’s learning approach is important as it is a key factor in any student’s educational journey and is known to be influenced by the learning environment. Understanding the factors which may predict students’ preference for a learning approach may also allow educators to better predict what types of adjustments to the learning environment may be needed. Many studies have reported the learning approaches in pre-medical/medical students in single-centre studies, with the dominant approaches being deep and strategic, with the surface approach being less common. Many studies also examine the impact of the approach to learning on academic performance, with deep and strategic approaches commonly associated with high performance and the surface approach more likely to be inversely associated with academic performance [8]. There is limited or conflicting evidence related to student characteristics associated with approach to learning, with some studies reporting differences across gender, educational background, and prior academic achievement [9–14].

## Methods

### Study aim

The primary research questions of this study were to determine the dominant approach(es) to learning science subjects in pre-medicine students at three different medical schools and to explore any associations that may exist between learning approach, gender, student educational background, English language proficiency, and academic performance through quantitative analysis. Using semi-structured interviews, the study also aims to develop insights into key factors shaping deep, surface and strategic learners’ approaches to learning.

### Study design

An explanatory sequential mixed-methods design was implemented in this research study in which pre-medicine students at three separate international medical schools were invited to complete an approaches to learning questionnaire (ASSIST). A small number of participants at each site were selected using purposive sampling and subsequently interviewed by a member of the research team using a semi-structured interview guide developed for the research study (Supplementary file 1).

### Study participants and setting

Pre-medicine students, 18 years of age or older, at three separate international medical schools located in Bahrain, Ireland and Malta were approached to participate in the study. Each of the medical schools actively recruits international students from many different regions, including Europe, North America, Asia and the Middle East. The participants, who were enrolled in a pre-medical course, were generally experiencing their first year of university studies following graduation from high school. The participants entered their respective programmes having completed a wide variety of high school qualifications including but not limited to the A-Levels, International Baccalaureate (IB), American High School Diploma (AHSD), Canadian High School Diploma (CHSD), Central Board of Secondary Examinations (CBSE), Bahrain Tawjihiya and Kuwait Tawjihiya. The foundation programme curriculum at the Bahrain and Dublin sites is equivalent, consisting mainly of traditional didactic teaching events and practical classes, with 30% of the assessment being continuous and the remainder being end-of-semester summative exams. The foundation programme at the Malta site also features traditional didactic instruction, hands-on practical classes, in addition to case-based tutorials and formative assessments for learning, with only summative exams contributing to students’ results. Students from all three sites are required to successfully pass all modules in the respective programmes before progressing to the medicine programme.

### Data collection and analysis

#### Quantitative

The instrument used in this study, the Approaches and Study Skills Inventory for Students, ASSIST [6], was primarily chosen because it measures all three approaches: Deep, Strategic and Surface. Its long form, comprising 52 items, has been utilised in numerous studies in a wide variety of higher education courses such as science [9, 15], engineering [16], occupational therapy [17] and also, of relevance to this study, in medicine [10, 18–31]. The ASSIST short form, comprising 18 items which are a subset of the original 52 items, measures deep, strategic and surface approaches and has been used in a variety of courses in higher education [32–37] as well as for the evaluation of approaches to learning in General Practitioners and Trainee GPs [38] which reported satisfactory internal reliability measures in the range of Cronbach’s α = 0.65–0.83. The short form ASSIST questionnaire was specifically selected for this study due to its low burden and satisfactory internal reliability measures.

Participants were invited to complete the short form of the ASSIST questionnaire [39] on paper at the end of a scheduled class during the second semester of the academic year of 2018/2019. Only students who agreed to participate in the study completed the study questionnaire and consent form, which were collected by a gatekeeper who was not a teacher of the students or a member of the research team to minimise the potential for coercion. Questionnaire data from each site were compiled with academic results when they became available, and participant identities were encoded in all data records by a gatekeeper at each site. The ASSIST responses, demographic data and academic performance were imported to Statistical Package for the Social Sciences, SPSS version 28 [40], cleaned, coded and checked for reliability by calculating the Cronbach alpha scores for each ASSIST scale (Deep, Strategic and Surface) and for normality with the Shapiro-Wilk test. The academic performance of participants in Bahrain and Dublin was also tested for normality using the Shapiro-Wilk test. Non-parametric tests (Mann-Whitney U test and Independent Samples Kruskal-Wallis test with Dunn’s post-hoc pair-wise comparisons) were used when investigating differences between groups in the ASSIST scales, and parametric tests (Independent samples t-test, and one-way ANOVA with Bonferroni-adjusted post hoc pairwise comparisons) were used when assessing academic performance across different groups. Using hierarchical linear regression analysis, predictors of academic achievement in the foundation programme were assessed with background factors entered into the model at step 1, and the assist scales added at step 2.

Participants were also assigned a single or combined preferred learning approach using a previously reported method [38]. On a scale from 6 to 30, participants with a single construct score 3 or more scale units above the other constructs were assigned that single construct as their preferred approach to learning. Participants for whom the highest scale score was less than 3 units greater than one other construct and 3 units or more greater than the other remaining construct were assigned combined constructs as the preferred approach to learning. Pearson Chi-Square tests were used to examine the distribution of participants in the preferred approach to learning across various groups.

#### Qualitative

Purposive sampling was used to select participants for an interview with the goal of obtaining a mix of participants which demonstrated a tendency for each of the three main approaches (either single or combined), and possessing different demographics and education backgrounds. One member of the research team, who was not directly involved in the teaching of the study participants, conducted the semi-structured interviews using a prepared question sheet with standardised probes (Supplementary file 1) designed to initially examine study behaviours before seeding reflective responses to their specific approaches to study. A total of 25 interviews were conducted, 8 in Bahrain, 7 in Malta and 10 in Dublin, all interviews were conducted face-to-face in English at each campus. The interviews lasted no longer than an hour, were recorded using a digital recorder and sent to a third-party transcription service for verbatim transcribing. Thematic analysis was conducted using a modified Braun & Clarke approach [41]. The transcripts were read by two of the study investigators before being uploaded to Dedoose, the online qualitative analysis platform. The two investigators responsible for qualitative analysis coded each interview transcript separately until coding was complete for all 25 interview transcripts.

The transcripts for the interviews of each learning approach category at each site were read and re-read by the investigators, who recorded their own personal notes and reflections. Transcribed notes along with field notes were subjected to line-by-line analysis by the investigators, with close attention paid to experiential claims, concerns, and understandings of the participants. Keywords, phrases and or descriptions from the participants were documented, as the authors reflexively engaged with the data. Convergence and divergence of data were noted, leading to the development of preliminary emergent themes. These themes were further interrogated and refined with reference to participants’ original words while also including the author’s collective interpretations.

## Results

### Quantitative analysis

All statistical analysis results not presented in tabular format within the results section can be found in Supplementary file 2. One hundred and fifty-nine pre-medicine students participated in the study, of which 59 were in Bahrain, 83 in Dublin, and 17 were located in Malta, representing response rates of 49%, 52% and 100% at each site, respectively. Each site had a similar gender balance, with approximately 60% of participants being female. Prior to joining the pre-med foundation programme, 27% of students completed other foundation or preparatory programmes, 19% a Tawjihiya qualification, 19% a American High School Diploma, 16% a Canadian High School Diploma, 12% the International Baccalaureate, and 7% another curriculum. The Central European Framework of Reference for languages (CEFR) English level of all participants showed that Dublin participants, had the highest level of English language proficiency, followed by Bahrain and then Malta. Academic achievement of participants upon entry to the foundation programmes was available for participants in Bahrain and Dublin. Participants who were judged to have presented entry scores which significantly exceeded the minimum entry requirements were categorised as high achieving, while those who just met or marginally exceeded admission entry requirements were classed as low achieving. Fifty-one per cent of Bahrain participants and 80% of Dublin participants were ranked as having high academic achievement upon entry. The participant characteristics are summarised in Table 1.

**Table 1:** Participant characteristics across sites

	Bahrain	Dublin	Malta^a^	Total
Gender	n (%)	n (%)	n (%)	n (%)
Male	24 (41)	32 (39)	7 (41)	63 (39)
Female	35 (59)	51 (61)	10 (59)	96 (61)
Education Background on Entry				
American High School Diploma	11 (19)	19 (23)	0 (0)	30 (19)
International Baccalaureate	11 (19)	8 (10)	0 (0)	19 (12)
Canadian High School Diploma	7 (12)	19 (23)	0 (0)	26 (16)
Prior Foundation Programme	14 (24)	29 (35)	0 (0)	43 (27)
Tawjihiya	12 (20)	1 (1)	17 (100)	30 (19)
Other	4 (7)	7 (8)	0 (0)	11 (7)
English language CEFR level				
B2	24 (41)	19 (23)	12 (71)	55 (35)
C1	19 (32)	19 (23)	5 (29)	43 (27)
C2	16 (27)	45 (54)	0 (0)	61 (38)
Academic achievement upon entry				
Low	29 (49)	17 (20)	-	46 (32)
High	30 (51)	66 (80)	-	96 (68)

^a^Data on academic achievement upon entry was not available for Malta site

### ASSIST instrument reliability

The reliability of the ASSIST (18-item) short form has been reported previously as having Cronbach’s alpha values in the range of 0.65 and 0.82 [32, 33], with the Deep scale showing lower alpha values by comparison to the other scales. The alpha values for the whole study sample were shown to be Deep = 0.51, Strategic = 0.73 and Surface = 0.71. The alpha values for the Strategic and Surface scales indicate good internal consistency; however, the Deep scale falls below the accepted minimum alpha threshold value.

### Predominant learning approaches

Medians and inter-quartile ranges (IQR) were calculated for the whole study sample and individual university sites separately. Whole sample median values for each scale were: Deep [23], Strategic [23] and Surface [19], Table 2. Deep and Strategic approaches to learning were identified as being more dominant compared to the surface approach at each of the sites.

**Table 2:** ASSIST scale values

	Deep	Strategic	Surface
	Median (IQR)	Median (IQR)	Median (IQR)
All sites	23 (21–26)	23 (19–26)	19 (15–22)
Dublin	23 (21–25)	23 (20–26)	20 (16–23)
Bahrain	24 (21–27)	21 (18–26)	19 (15–22)
Malta	24 (22–26.5)	25 (20–26.5)	13 (10–17.5)

**Fig. 1:**
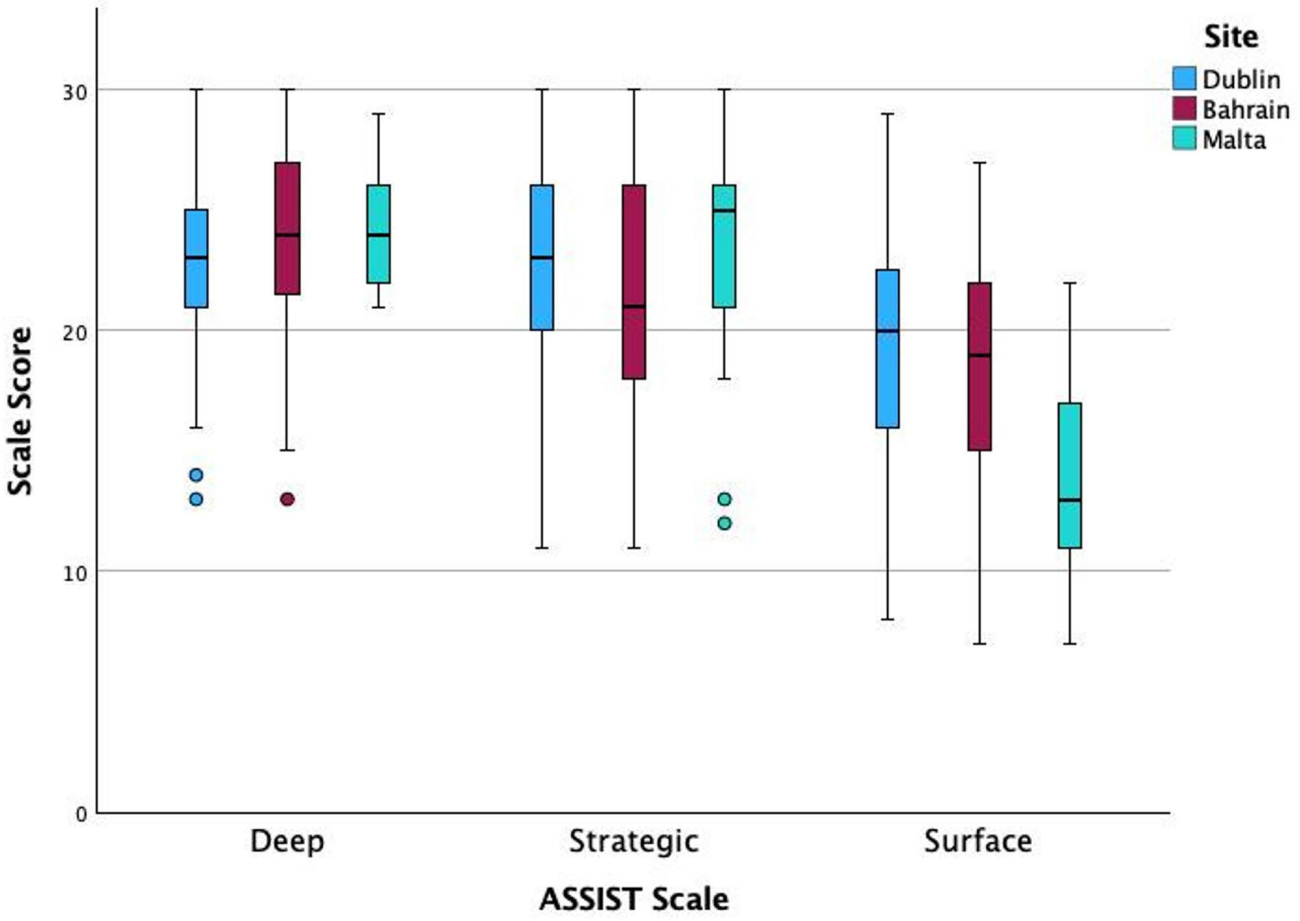
Boxplots of ASSIST deep, strategic, and surface scale scores by site (Dublin, Bahrain, Malta)

Shapiro–Wilk tests indicated deviations from normality for all three ASSIST scales (Deep: *W* = 0.971, *p* = 0.002; Strategic: *W* = 0.965, *p* < 0.001; Surface: *W* = 0.980, *p* = 0.024; *N* = 159). Kruskal-Wallis tests showed a significant effect on the Deep scale, χ^2^ (2, *N* = 159) = 7.927, *p* = 0.019, and Surface scale χ^2^ (2, *N* = 159) = 16.853, *p* < 0.001, between the three different sites, but not on the Strategic scale χ^2^ (2, *N* = 159) = 2.391, *p* = 0.303. Dunn’s post-hoc tests with Bonferroni adjustment indicated that Dublin had lower Deep scores than Bahrain (*z* = −2.51, adj. *p* = 0.036, *r* = − 0.20). Dublin-Malta (*z* = − 1.90, adj. *p* = 0.172, *r* = − 0.15) and Bahrain–Malta (*z* = − 0.29, adj. *p* = 1.000, *r* = − 0.02) contrasts were not significant. On the Surface scale, Malta had lower scores compared to both Bahrain (*z* = 3.48, adj. *p* = 0.001, *r* = 0.28) and Dublin (*z* = 4.09, adj. *p* = 0.000, *r* = 0.324) and no significant difference was observed for Bahrain-Dublin (*z* = 0.767, adj. *p* = 1.0, *r* = 0.06). Fig. 1 displays a boxplot chart of Deep, Strategic and Surface scale scores by site (boxes = IQR, line = median, whiskers/outliers shown).

### Preferred approach to learning

All participants were categorised as having a single or combined preferred approach to learning based on scores in the ASSIST scales using a method previously reported [38]. The preferred approaches to learning categories are: Deep, Strategic, Surface, Deep-Strategic, Deep-Surface, Strategic-Surface, No preference. The distribution of the preferred learning approach of students in the study sample is presented in Table 3. The dominant preferred approaches to learning of participants in the study sample were Deep (21%), Strategic (21%) and Deep-Strategic (29%).

**Table 3:** Participants’ preferred approach to learning

Site	All sites	Bahrain	Dublin	Malta
	N (%)	N (%)	N (%)	N (%)
Deep	33 (21)	14 (24)	13 (16)	6 (35)
Strategic	33 (21)	6 (10)	21 (25)	6 (35)
Surface	8 (5)	3 (5)	5 (6)	0 (0)
Deep-Strategic	46 (29)	20 (34)	22 (27)	4 (24)
Deep-Surface	12 (8)	5 (8)	7 (8)	0 (0)
Strategic-Surface	7 (4)	3 (5)	4 (5)	0 (0)
No Preference	20 (13)	8 (14)	11 (13)	1 (6)

### Associations between demographic factors, background and approaches to learning

Considering the observed significant differences between Malta and the other sites on two of the ASSIST scales, and in addition to Malta following a different curriculum and assessment strategy than both Bahrain and Dublin, participants at the Malta site were excluded from all quantitative analyses assessing associations between student demographics, background, and approaches to learning.

#### Gender

Mann-Whitney U tests for the ASSIST scales across gender groups returned values > 0.05 for each of the Deep, Strategic and Surface scales, indicating that there is no difference in the distribution of any ASSIST scale according to gender (Deep: *U* = 2056, *z* = −1.48, *p* = 0.140; Strategic: *U* = 2349, *z* = −0.25, *p* = 0.805; Surface: *U* = 1975, *z* = −1.81, *p* = 0.070; *N* = 142).

#### English language proficiency

**Fig. 2:**
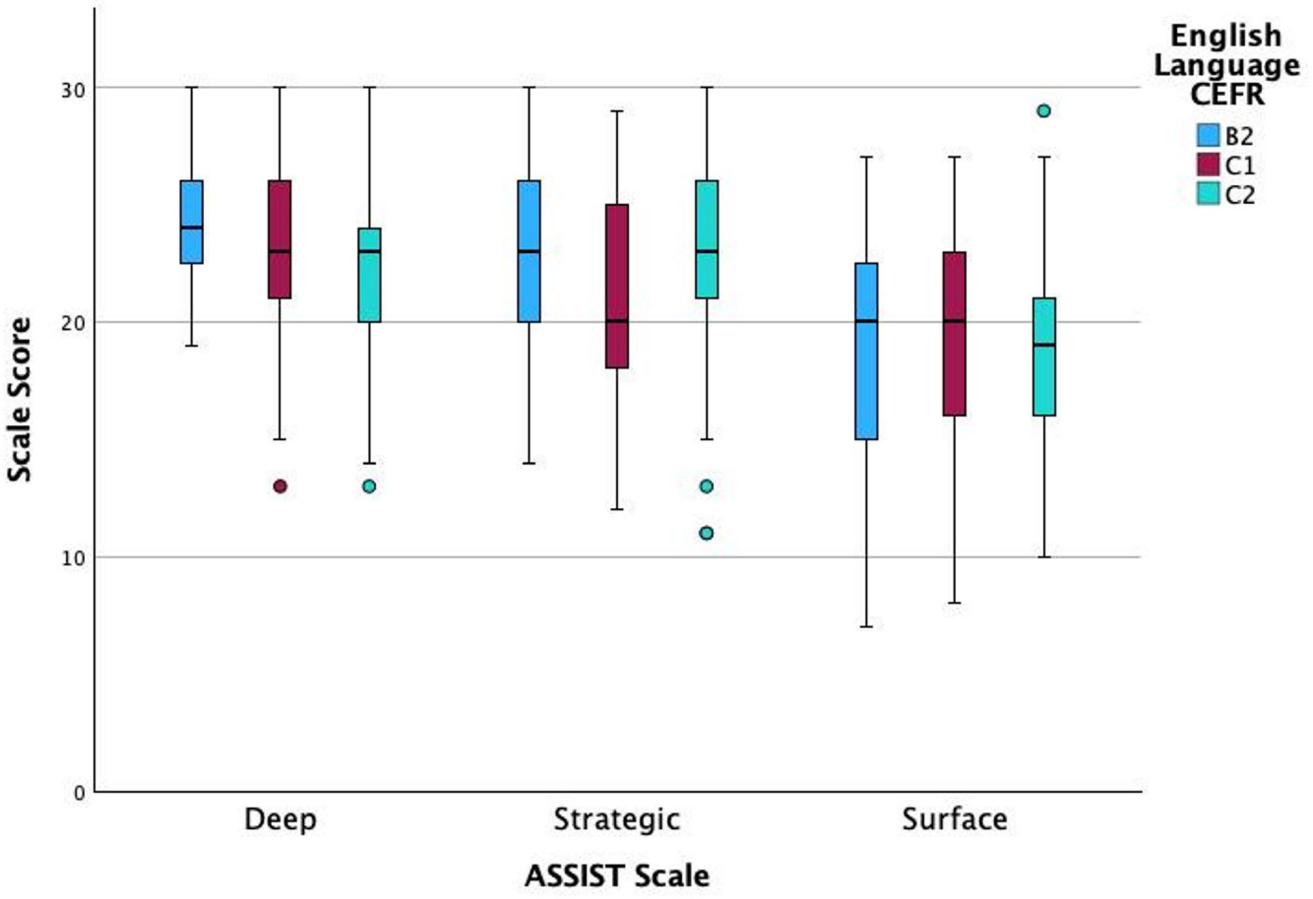
Boxplot of ASSIST deep, strategic and surface scale scores by English language CEFR levels (B2, C1, C2)

A Kruskal-Wallis test showed an effect of English Language CEFR level on the Deep scale, χ^2^ (2) = 7.56, *p* = 0.023, but not on the Strategic scale, χ^2^ (2) = 5.51, *p* = 0.064, or the Surface scale, χ^2^ (2) = 1.43, *p* = 0.488, (all *N* = 142). Dunn’s post-hoc test with Bonferroni adjustment indicated that B2 level students scored higher than C2 students (*z* = 2.73, adj. *p* = 0.019, *r* = 0.23). Differences between C2-C1 (*z* = 1.41, adj. *p* = 0.158, *r* = 0.12) and C1-B2 (*z* = 1.128, adj. *p* = 0.777, *r* = 0.10) were not significant (Fig. 2).

#### Education background

**Fig. 3:**
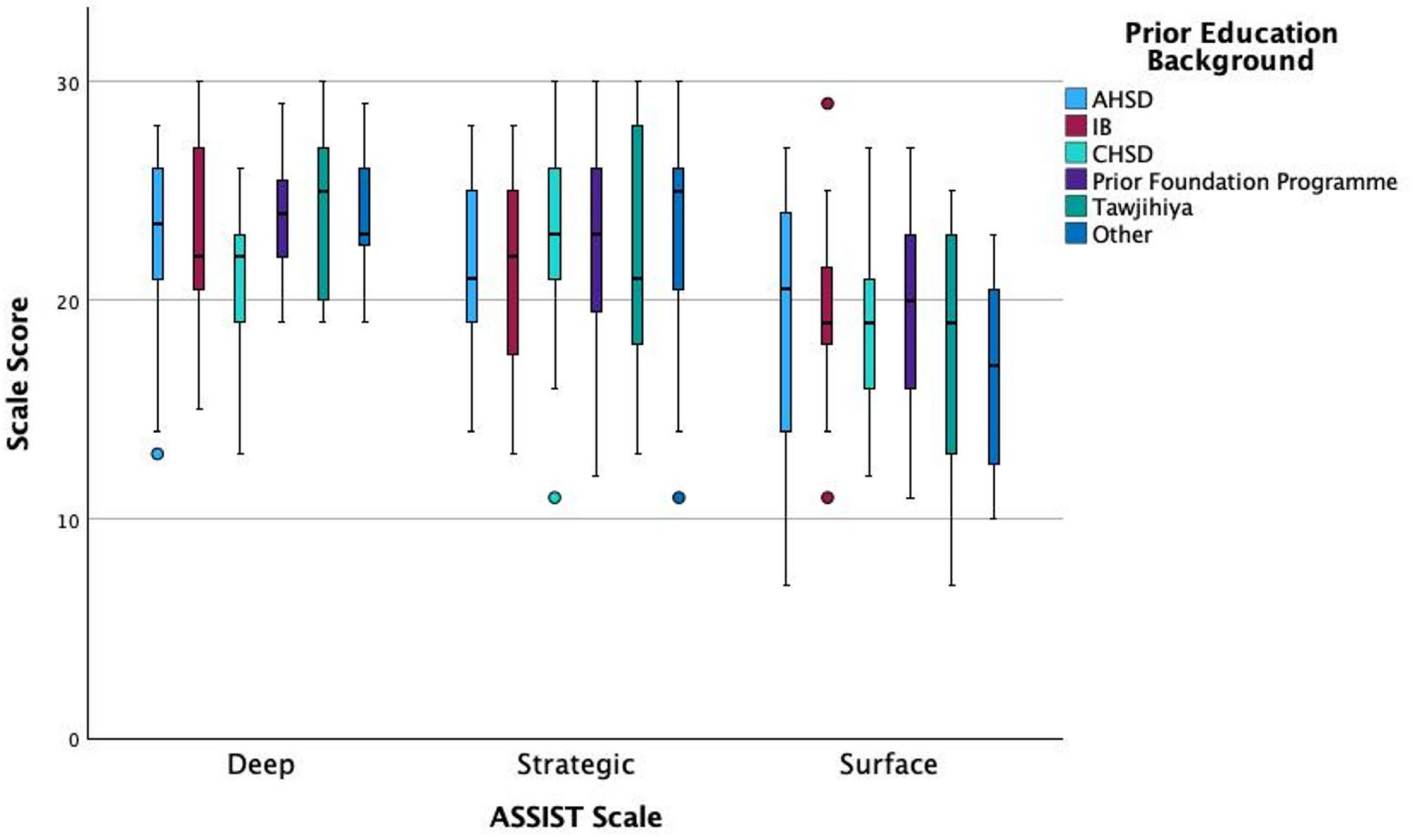
Boxplots of ASSIST deep, strategic and surface scales by prior education background

Participants were grouped according to the type of curriculum studied immediately prior to joining the pre-medical foundation programme (e.g. AHSD, CHSD, IB, Prior preparatory/foundation programme, Tawjihiya, and other). Curricula which only featured a small number of data points (Irish Leaving Certificate, A-Levels, Norwegian High School Diploma, CBSE) were categorised as other curricula. Kruskal-Wallis tests showed a significant effect of prior education background on the Deep scale, χ^2^ (5) = 13.87, *p* = 0.016, but no effect on the Strategic scale χ^2^ (5) = 2.56, *p* = 0.768, or Surface scale, χ^2^ (5) = 4.19, *p* = 0.523, (*N* = 142). Dunn’s post-hoc comparisons with Bonferroni adjustment indicated students who had previously attended CHSD scored lower on the Deep scale than students who had attended another preparatory/foundation programme (*z* = −3.321, adj. *p* = 0.013, *r* = −0.279). All other pairwise interactions on the deep scale were not significant after adjustment (Fig. 3).

#### Academic achievement on entry

The Mann-Whitney U tests showed no effect of academic achievement on entry on any of the three ASSIST scales (Deep: *U* = 2206.5, *z* = −0.11, *p* = 0.910; Strategic: *U* = 2425, *z* = 0.84, *p* = 0.403; Surface: *U* = 2423.5, *z* = 0.83, *p* = 0.407; *N* = 142).

### Predictors of academic performance in the pre-medical foundation programmes

Academic performance data were available for all participants from the Bahrain and Dublin sites. Participants at these sites follow the same curriculum and sit the same summative assessments. The Shapiro–Wilk test indicated that academic performance scores did not significantly deviate from normality, *W*(142) = 0.98, *p* = 0.090.

#### Site, gender and English language proficiency

Independent samples t-tests showed no difference in academic performance during the foundation programme between Dublin and Bahrain sites, Welch’s *t*(125.46) = 0.772, *p* = 0.441, and by gender, Welch’s *t*(129.40) = −1.439, *p* = 0.153. A one-way ANOVA showed English language CEFR levels also did not impact academic performance, *F*(2,139) = 1.605, *p* = 0.205, *η*^2^ = 0.023 (*N* = 142).

#### Education background

Prior education background was found to influence academic performance by a one-way ANOVA test, *F*(5,136) = 2.94, *p* = 0.015, *η*^2^ = 0.097. The Games-Howell pairwise test identified that Tawjihiya students had significantly higher academic performance than IB students (Tawjihiya: M = 74.15, SD = 11.73, *n* = 13; IB: M = 60.32, SD = 10.42, *n* = 19; mean difference = 13.84, adj. *p* = 0.024; Hedges’ g = 1.23). No other pairwise differences were significant after adjustment.

#### Academic achievement on entry

Unsurprisingly academic performance was higher for students with high levels of academic achievement on entry (*n* = 95, *M* = 68.29, *SD* = 11.20) compared to those with low levels of academic achievement (*n* = 47, *M* = 59.34, *SD* = 10.72), Welch’s *t*(95.48) = 4.61, *p* < 0.001, mean difference = 8.95, 95% CI [5.10, 12.81], Hedges’ *g* = 0.81, 95% CI [0.44, 1.17].

#### ASSIST scales

Pearson correlations were calculated for all three ASSIST scales and academic performance in the foundation programmes (*N* = 142). Academic performance was positively correlated with the Strategic approach, *r* = 0.23, *p* = 0.007, but not with Deep, *r* = 0.08, *p* = 0.33, nor Surface, *r* = − 0.16, *p* = 0.06. Among the ASSIST scales, Strategic correlated positively with Deep, *r* = 0.21, *p* = 0.013, and negatively with Surface, *r* = − 0.21, *p* = 0.013; the Deep–Surface correlation was not significant, *r* = 0.06, *p* = 0.47. Although statistically significant, these effects were small in magnitude, indicating weak associations that warranted further analysis.

The associations between the ASSIST scales and academic performance were examined using multiple linear regression. This model was run to confirm whether the ASSIST scales contribute to academic performance beyond other demographic and background factors. Model 1 (Site, Gender, CEFR, Prior Education Background, Academic Achievement on Entry) was significant, *R* = 0.419, *R*^2^ = 0.176, adj. *R*^2^ = 0.145, *F*(5,136) = 5.79, *p* < 0.001, explaining roughly 14.5% of the variance in Academic Performance. Within Model 1, Academic Achievement on Entry was a positive predictor (*B* = 8.04, β = 0.32, *p* < 0.001), and Prior Education Background, a positive predictor (*B* = 1.51, β = 0.20, *p* = 0.016); Site, Gender, and CEFR were not significant. Model 2 added the ASSIST scales (Deep, Strategic, Surface) and improved fit, *R* = 0.477, *R*^2^ = 0.228, adj. *R*^2^ = 0.182, *F*(8,133) = 4.91, *p* < 0.001, yielding Δ*R*^2^ = 0.052 over Model 1, explaining 5.2% additional variance. In the final model, Academic Achievement on Entry (*B* = 8.05, β = 0.32, *p* < 0.001) and Prior Education Background (*B* = 1.26, β = 0.16, *p* = 0.042) remained significant positive predictors. However, none of the ASSIST scales reached conventional significance (Deep: *B* = 0.23, β = 0.07 *p* = 0.42; Strategic: *B* = 0.43, β = 0.16, *p* = 0.053; Surface: *B* = − 0.29 β = −0.12, *p* = 0.162). Notably, the bivariate correlation between the Strategic scale and Academic Performance (*r* ≈ 0.23) attenuated and lost conventional significance once we controlled for prior achievement and background factors in the regression, indicating the influence of covariates.

### Qualitative analysis

Twenty-five participants from the three campuses took part in the qualitative phase of the study. Once transcription was completed, the deductive coding approach was employed, and the key findings are described below.

One important finding was the tendency for students to adopt a subject-specific approach to learning even when they were utilising a preferred learning approach. They described a tendency to flip-flop between learning approaches depending on the science subject they were learning.

*“I’d say it probably depends on the subject. Does that make sense. For physics*,* sort of calculation that type of thing*,* I tend to probably approach towards the strategic. Does that make sense. I’m very much studying for the exam. I have a very organized way that I’m preparing for the exam. For things that I’m really interested in*,* and I think that I can cover a lot of ground in*,* I tend to be a deep learner.” (ASSIST-07-DUBLIN)*.

*“I feel I’m a bit of the deep learner and mostly strategic learner but just a bit of a deep learner. On subjects that*,* I can like*,* I feel passionate about.” (ASSIST-02-DUBLIN)*.

*“I try to do strategic mix between. I preferred deep when I was really interested in the subject. So*,* if there’s something I come across that I’ve genuinely really like I’ll try to go to that and find more information about it. But if it’s something that I just*,* that I understand I’ll move on. But as far as the course I have to know what it’s going to come in the exam and I have to do well. I’ll just learn over that. But if I like something I’ll go deep.” (ASSIST-02-BAHRAIN)*.

During the interviews, students were given the opportunity to reflect more deeply on the notions of deep, strategic, and surface approaches to learning. For each learning approach, we identified key characteristics contributing towards defining the image of each type of learner. The findings are detailed in Table 4.

**Table 4:** Qualitative analysis summary results table

THEME	SUBTHEME	EXPLANATION	QUOTE
DEEP	Embedding science into medicine	Embedding science into medicine gave participants an opportunity to make associations with the science topics being taught to clinically related areas such as chemotherapy and cancer.	*“For things that I’m really interested in. And I think that I can cover a lot of ground in. I tend to be a deep learner…To be honest I had a really big nerd moment when you started learning about chemotherapy drugs. There was a 40-minute Wikipedia like researched literature dive into doxorubicin because there was one sentence about cardiotoxicity on the slide.” (ASSIST-07-DUBLIN)*
	Connecting concepts	Combining similar subject matter, resulting in links being built between other subjects. An important part of this process was to identify the right resources to facilitate this.	*“I think it’s kinda enough if I go through it and go through the book but when I started going through the book*,* I started linking information a bit more together. And this is important specially for writing essays. It’s not about focussing on a slide as a specific topic but it’s about the whole topic. So*,* if I read the book*,* I can link ideas together much better.” (ASSIST-06-MALTA)*
	Seeking greater meaning	This notion was built around wanting to build depth in knowledge to improve understanding. An important element contributing towards this was to establish accuracy of lecture content through their own fact-finding research leading to deeper insight into the lecture content.	*“Because I always want to know more. I always want to know the details of about things like even when I was in school. In the university or in school if the doctor said something I want to know more about it. I want to know how does it work? Why does it work like this? So*,* and I think it’s useful for the extra information in the summative (exams) for example.” (ASSIST-01-BAHRAIN)*
SURFACE	Fear of failing	The key driver in adopting a surface approach to learning was the fear of failing. At the start of the pre-medicine course there is no formal handover from secondary school to higher education, and thus, participants are left trying to identify the right learning approach.	*“Actually I want to learn but*,* and I want to guarantee my future because if a medical student*,* they fail this foundation year*,* they will return back…. You change the university or you change the country. Very like messy process. So I want to learn but I have the pressure that I should guarantee my future.” (ASSIST-01-MALTA)*
	A passive approach to learning	Passive learning was a common activity among participants who spent a lot of their time reading through lecture content, a recognised passive learning activity. Surface learners also found themselves dwelling far too long on difficult content and failing to identify and fill gaps in their knowledge.	*“I just sat and opened my laptop and I used to print the lectures*,* so I just tried to go through everything…The first semester I used to only read.” (ASSIST-01-DUBLIN)*
	Used memorisation as a study aid	Memorisation of content was adopted as a learning strategy for many surface learners and essentially used repeatedly until content was ‘remembered’. This way of learning was unsatisfactory for some participants as it was not useful for long-term recall of essential content, it was also laborious and time-consuming.	*“For me as I said first*,* I was literally memorizing everything even like the diagrams that like how I can see the diagrams that has chemical equations in a biology lecture. I was memorising them but when I saw the exam*,* I realized that they are like asking on the main points or always that always comes in the main point…Yes*,* the material is way more than school. But at school*,* they used to ask us about every single thing. That’s why I started doing memorizing every single thing. At first*,* I was like OK so I’m gonna memorise everything and it appeared overwhelming. Like after(wards) I know that they want just us to understand and memorise and so it’s still way more than the school right.” (ASSIST-06-BAHRAIN)*
STRATEGIC	Organised their learning	Being organised meant autonomously locating appropriate software such as a suitable scheduling tool to account for all time spent on studying and alternative tools to help organise lecture content to facilitate learning. Grouping lectures on the same subject helped learn content holistically and writing summaries using their own words helped with knowledge acquisition.	*“There is an application called Anki on the laptop. It’s a flashcards application. So*,* I write*,* while I’m going through the lecture*,* I write questions and then I write the answer to them. And later on*,* I go through the questions and answers…Like for example of if there is a lecture if there is a 15-lecture exam then I have to go through each lecture at least three or four times. Okay so there is a plan that I have in my notes that says I went through this lecture these many times and the last time I went through it was for example 15 March” (ASSIST-04-BAHRAIN)*
	Were proactive learners	Focusing on learning outcomes and grouping slides to answer learning outcomes was a key step towards being proactive, as was identifying topics that could not be easily understood by reviewing lecture content alone. Locating suitable alternative resources such as videos and eBooks to improve understanding was a necessary step in filling gaps left after reviewing lecture content.	*“So*,* I see the number of slides and then I go through the outcomes and then I write the outcomes. So basically*,* I’m doing what the embryonic origin then I’m doing what’s the role of this in the body and how this influence the body and what is the diseases. So*,* I have four or five categories. And then I see okay I go to the lecture I say okay embryonic. I don’t understand what this is. What is this. I don’t understand. So*,* I put a star and I go Google it. I see videos first. I see video maybe 10 minutes. I don’t understand. I don’t to waste time on it. I go and read. I did not understand what the content is saying. I go I e-mail the lecturer*,* or I go see them. This is my method now*,* not in the last semester…So it saves time for me in an order rather than going back and studying all and then identifying like the other subjects…I did some exams before it just continuous assessment and I did good. I got a really a good grade.” (ASSIST-01-DUBLIN)*
	Used academic performance as a driver	The importance of aligning their approach to learning with the assessment method which also guaranteed a strong academic performance.	*“Because my end goal in first year since I came in knowing a lot of the stuff already was to just make sure that I knew what was needed for the exams and that I knew the material that was able*,* I was able to do my best. I was able to show my understanding of them and to learn it in such a way that I would be able to help me in my later years not in a way that I would forget it like a month later but because that’s what that was what I did all of high school.” (ASSIST-08-DUBLIN)*

Those who adopted a deep learning approach embedded science into medicine, connected concepts, and sought greater meaning.

Others using a surface learning approach were afraid of failing, adopted a passive approach to learning, and employed memorisation as a study aid.

Participants applying a strategic approach to learning optimised their learning approach by concentrating on organising their learning, became proactive learners and used academic performance as a driver.

In summary, the three main approaches to learning were described by the majority of the interviewees. Most did not adhere to a single approach; instead, they used a mixture of approaches to ensure that they had a firm appreciation of lecture material. However, the single preferred approach that stood out in our interviews was the surface approach, which was adopted by those who struggled with time management and used reading alone as their passive learning method.

## Discussion

The goal of our investigation was to explore in detail the approaches to learning adopted by foundation programme/pathway pre-medicine students at three medical schools across the Middle East and Europe. This is the first study to utilise a mixed-method design and the ASSIST instrument on a student population across three international medical schools.

### Predominant approach to learning

The quantitative results from the ASSIST instrument show significant agreement with many of the previous studies featuring medical students. The students at all three universities show a preference for adopting a strategic approach (21%), a deep approach (21%) or a combination of deep and strategic approaches (29%). Ward et al. [24] reported that medical students studying anatomy had a strong preference for either deep or strategic learning, while Reid et al. [20] reported that medical students exhibited a predominance for strategic and deep learning when studying biology and disease. Chonkar et al. [31], who surveyed clinical students during an obstetrics and gynaecology rotation, also reported deep and strategic approaches as most dominant. While the occasional exception exists, in general, the literature suggests that medical students at both preclinical and clinical stages have strong tendencies towards deep and strategic learning, which may become even more pronounced during postgraduate training [27]. The most striking observation regarding the approach to learning preference of the pre-medicine student population in our study is a very similar distribution of preferred approach to learning to that published by Curtis et al. in 2018 for a sample of general practitioners (GPs) and GP trainees in the UK [38]. While it is well accepted that the learning environment has a profound impact on learning approach, we see a similar distribution of learning preference in two different groups at very different stages in their medical education journey.

Deep and strategic approaches also appear to be preferred in student populations from other health science programmes, including occupational therapy and physical therapy [17, 35]. These approaches have also been reported as predominant in a study by Ballantine et al., which assessed students in accounting and business studies programmes [42]. On the other hand, there are limited studies that report contrary results in which the surface approach to learning is most prevalent [9, 32], two of which feature students studying plant biology or introductory chemistry in multiple pathway class groups.

It is reasonable to expect that the majority of students in health professions programmes, including medicine, have sufficient intrinsic motivation and interest in the subjects of study to allow for deep and strategic approaches to dominate. The presence of intrinsic motivation may contribute to the existence of little evidence for surface approaches within health professions programmes, despite the presence of factors, such as demanding workloads and rigorous assessment schedules, that can give rise to surface approaches to learning.

### Cross-site differences in ASSIST scales

It is well accepted that a student’s approach to learning is a product of their perception of and response to their learning environment. While the predominant approaches to learning at each site in our study were identified as Deep and Strategic, statistically significant differences that were observed in the values of the Deep scale between Dublin and Bahrain were small and not likely to have practical implications. The Surface scale differences between Malta and both Dublin and Bahrain were small to moderate, indicating meaningful differences but not large. A large multi-centre study examining occupational therapy students in Norway, Hong Kong, Singapore and Australia [43] observed no difference in the Deep scale across sites, but significant differences in the Strategic and Surface scales were observed between sites, with particular subscales contributing to these differences. In this study, the authors cited differences in mode of delivery (PBL, traditional instructor-centred approach) as potentially key to explaining the observed differences. The Bahrain and Dublin sites share many common features within their curriculum, delivery and assessments that Malta does not share. While it is accepted that curriculum content, the manner of delivery, and assessment contribute to approaches to learning, given the small to moderate effect sizes or modest differences between sites, we do not make strong causal claims regarding the impact of the learning environment between sites on the observed differences.

### Student demographic and education background factors impacting approaches to study

Although there are reports in the literature of differences in approaches to learning across gender [9–12], we did not observe differences according to gender on any of the ASSIST scales in our study. Of the other student characteristics which we assessed, academic achievement on entry was also found to have no influence on students’ approach to learning, in contrast to reports by Valadas et al. [14] who observed that prior academic achievement predicted approach to learning. Deep and strategic scales were positively correlated with prior academic achievement, and the surface scale was negatively correlated. A possible reason that we did not observe a similar association may be the diverse nature of the education qualifications that students present upon entry to the foundation/pathway programmes. While every effort is made to equilibrate the standard of each qualification accepted for admission, each qualification can have significant variation in the proportion of standardised assessment that contributes to the final outcome of the education qualification.

Rather unexpectedly, students with lower levels of English language proficiency had higher deep scale mean values compared to students with higher English language proficiency. Contrary to our findings, a small number of studies that explored the issue reported that lower levels of English language competence predict an increased surface approach to learning rather than deep approach [44, 45]. In the current study, students with lower levels of English proficiency who are enrolled in the foundation/pathway programmes typically come from government schools where Arabic is the primary language of instruction. Very often, these students also tend to be high academic achievers in high school and the recipients of government scholarships to study medicine, with high levels of intrinsic motivation, which may contribute to this observation.

A significant difference on the Deep scale between students who have different prior educational backgrounds (CHSD and prior foundation programme) cannot be easily explained. There is little evidence in the literature to suggest that educational experiences in school can moderate approaches to learning at university. In the past, teaching and assessment policies that promoted surface learning, such as rote memorisation, were widespread, but in recent years, many national and regional curricula (including the Canadian provinces), have been revised to adopt competency-based outcomes and assessments that encourage deeper learning [46]. Perhaps the observed difference might be a product of variations in maturity and transition to third-level learning that have been reported in other studies [27, 47]. The CHSD students were experiencing post-secondary learning for the first time, while the prior foundation programme group had already completed a post-secondary academic programme. While the observed difference between CHSD and prior foundation programme groups was significant and strong, it should be noted that no other differences between different education backgrounds were detected, suggesting that approaches to learning can be adapted quickly. Several longitudinal studies that tracked students during university programmes, have found that the approach to learning can evolve as they progress through the programme and encounter changes in content, workload, delivery, and assessment [48–50]. It is reasonable to expect that targeted pedagogy and assessment in the foundation programmes have likely shaped approaches more than the students’ pre-university background, allowing little “carry-over” from prior school systems.

### Approaches to learning and academic performance

Significant positive associations between academic performance and the strategic scale are common observations reported in other medicine-based studies [21, 24, 25, 30]. Studies have less frequently identified positive correlations for the deep approach and a negative correlation for the surface approach with academic success. In our study, the Pearson correlation results suggested the presence of a weak correlation between the Strategic scale and academic performance; however, confirmatory analysis showed this correlation attenuated when other demographic and education background factors were controlled for. As educators, we tend to emphasise the importance of being a deep learner, and promote the idea that a deep approach will transform into academic success. However, there is significant evidence indicating that being a strategic learner is just as likely to be associated with academic success [13].

### Qualitative insights

The short form ASSIST questionnaire revealed the approaches to learning adopted by foundation/pathway pre-medicine students at three different medical schools, but the more detailed responses from the interviews have provided insights into the adaptable nature of students’ approaches to learning. While the prevalent approaches were identified as deep and strategic, students frequently cited their willingness to adapt depending on the subject, content or assessment demands. This is an observation cited by Marton and Saljo, and Entwistle and Ramsden in their work on the development of the ‘approaches to learning’ model [1, 3]. Our analysis suggests that students with a predominantly strategic approach gained the most from working autonomously and utilising a number of resources and software tools to aid with the organisation of study material and time keeping. Those who identified as primarily deep or surface learners but without a strategic approach found themselves struggling to achieve the level of required knowledge to meet course assessment demands, while surface learners engaged primarily in passive learning activities. The activities often cited in strategic approaches were generally components of self-regulated learning (SRL), which have been shown to be not only positively associated with academic performance but also a teachable skills set rather than an innate trait [51, 52]. There is conflicting evidence in the literature to show that changes in a medical curriculum designed to move students away from surface approaches are effective [13]. Perhaps what is needed to guide students away from surface approaches and towards more successful strategies is not changes to the learning environment but structured training in self-regulated study behaviours.

## Limitations

This study has several limitations. It used a cross-sectional design and a self-report instrument to measure approaches to learning, which is vulnerable to response biases. Notably, the Deep scale showed low internal consistency (α ≈ 0.51), limiting confidence in results concerning this construct. The Malta cohort was small (*n* = 17) and homogenous in nature, and was thus excluded from most quantitative associations due to curricular differences, which reduced the generalisability of our results. This also reduced the sample size and, hence the power of our analysis, especially for group comparison analyses, which had a larger number of groups, thus increasing the chances of not detecting small effects. Since the interviews were conducted in English, this may not have captured the full range of perspectives, particularly for students with lower language proficiency or cultural differences. Data came from a single academic year and a specific pre-medicine context, so findings may not extend to other cohorts or programmes.

## Conclusions

Across three international pre-medicine cohorts, students most commonly reported deep and strategic approaches; however, interview data showed that they adapted to subject matter and assessment demands rather than adhering rigidly to a single approach. Between-site differences in approaches were limited in magnitude, and previously reported commonly observed associations between specific approaches and academic achievement were not confirmed to exist in this study. Rather than attempting to shift students toward a singular “ideal” approach, educators may achieve more by supporting self-regulated study behaviours (planning, organisation, resource use) across the cohort, designing assessments and feedback that reduce unhelpful surface tactics, and targeting transition supports for students in the first year of a new programme whose backgrounds or language proficiency place additional demands on their existing study skills. Longitudinal, multi-cohort studies that incorporate more reliable measurement of the Deep scale, examine assessment design and learning-environment features, and test targeted support interventions (e.g., self-regulated learning skills) are warranted to clarify potential associations with learning approaches in early medical education.

## Supplementary Information

Supplementary Material 1.

Supplementary Material 2.

## Data Availability

All available datasets supporting the findings of this study are available upon reasonable request from the corresponding author [DG].

## Abbreviations

AHSD: American High School Diploma
ASSIST: Approaches to study skills inventory in students
CBSE: Central Board of Secondary Examinations
CEFR: Central European Framework of Reference for languages
CHSD: Canadian High School Diploma
IB: International Baccalaureate
