# Supplementary material for: Approaches to learning in pre-medicine: a multi-university mixed-methods study
Source: BMC Med Educ. 2025 Nov 19;25:1622. doi: 10.1186/s12909-025-08228-x (PMC12628898; doi:10.1186/s12909-025-08228-x)
Supplement: Supplementary file 1 — Supplementary Material 1. [file 12909_2025_8228_MOESM1_ESM.docx]

**Approaches to Learning Project: Instructions for Interviewers**_________________________________________________________

**A Prior to Interview**
Before the start of the interview:

You should know the participant’s full name and their allocation (deep, superficial or strategic)

Digital audio recording equipment should be ready to record (test this before you begin).

**B Interview - Introductory script**

This script outline below should be used to set up the interview and to ensure that the interviewee is comfortable and ready to start.

Hi, thank you for agreeing to participate in this interview. My name is (staff full name) and I am part of the research team working on this project.

- You may remember, this interview is part of ongoing research exploring approaches to learning science subjects during the first year of medical school.
- When I ask you a question there are no right or wrong answers – we are interested in your opinions and your experience.
- Please feel free to interrupt if you do not understand the question fully or if you need to ask me a question.
- I will be recording this interview and I will be taking notes from time to time.
- I would like to emphasise that the information collected is completely confidential.
- Your responses are anonymised, and you will not be able to be identified from the data.
- The interview should not take any longer than 1 hour.
- Do you have any questions before we start?
- **I will start the recorder now**

**C Approaches to Learning: Interview Probes**_______________________________________________________________

1. **Can you tell me about your experience as a Foundation Year student, so far?**

- How have you adjusted to the new environment of university?
- How have you found making new friends?
- Can you explain whether you feel you fit in (or not) at the university / in FY?
- How have you found the volume and difficulty of the material so far?
- … Can you give me any examples to explain that?

1. **Can you explain to me how you approach learning in Foundation Year?**

- What do you do while you are in a science lecture/tutorial/practical teaching session?
- Describe how you schedule your learning or study time.
- On average how many hours do you study daily (outside the classroom)? Do you study every day? What about the weekend?
- Describe what type of pre-class preparations you undertake.
- If you make your own notes, describe the process of preparing them, what you produce and how you use them.
- Describe any additional learning resources you use them for learning.
- If you use online videos, describe how you use them for learning.
- If you use the lecture recordings, describe how you use them (lecture capture if applicable).
- Describe any self assessment techniques you use to measure your learning?

1. **Has the way you study science subjects changed since you came to Foundation Year from secondary school?**

- How?
- … Can you give me an example?

1. **Based on answers given in the survey you seem to adopt a Superficial/Deep/Strategic learning approach such as ………Why do you think you tend to use this approach?**

- Hard or more work, learning different, support –teachers, friends and family?

Volume of material, the way the subject is the taught, the nature of the assessment

Give examples

1. **Does the way the subject is taught affect the learning approach you use?**

- How the teacher teaches. The size of the class.
- … Can you give me an example?

1. **Does the nature of the assessment impact on your learning approach?**

- If yes, why, if no, why?
- … Can you give me an example?

1. **What is your primary focus when you are learning a subject?**

- Is it your own short/long term achievement goals?
- Is it the learning outcomes?
- Or the learning situation?
- … Can you give me an example?

1. **Do you think there is anything that you could be doing to improve your approach to learning science subjects?**
2. **Can you please review this sheet and identify the phrases which you consider are representative of the main motivations which drive your learning in general?**

Satisfaction while learning new things (Intrinsic: To know)

To enable me to enter the job market in a field that I like. (Extrinsic: Identified)

To obtain a more prestigious job later on (Extrinsic: Regulated)

Satisfaction to accomplish difficult academic activities (Intrinsic: To accomplish)

To show myself that I am an intelligent person (Extrinsic: Introjected)

In order to have a better salary later on (Extrinsic: Regulated)

For the "high" feeling that I experience while reading about various interesting subjects (Intrinsic: Experience stimulation)

Honestly, I don't know; I really feel that I am wasting my time in the Foundation Year (amotivated)

1. **Is there anything else you might like to add which may be important about the way you approach learning in the Foundation Year that I haven’t asked you about?**

**Concluding remarks**

If you have no further questions, I will end the interview now. Thank you for your time.

It is much appreciated.
