# Supplementary material for: Approaches to learning in pre-medicine: a multi-university mixed-methods study
Source: BMC Med Educ. 2025 Nov 19;25:1622. doi: 10.1186/s12909-025-08228-x (PMC12628898; doi:10.1186/s12909-025-08228-x)
Supplement: Supplementary file 2 — Supplementary Material 2. [file 12909_2025_8228_MOESM2_ESM.docx]

Table S1 – Normality test for Deep, Strategic and Surface scales for all sites

|  | Shapiro-Wilk | | |
| --- | --- | --- | --- |
|  | Statistic | df | Sig. |
| Deep | 0.971 | 159 | 0.002 |
| Strategic | 0.965 | 159 | <0.001 |
| Surface | 0.980 | 159 | 0.024 |

Table S2 – Kruskal-Wallis test for distribution differences across sites (Bahrain, Dublin and Malta)

|  |  | Kruskal-Wallis | | |
| --- | --- | --- | --- | --- |
|  | N | χ^2 a^ | df | Sig. |
| Deep | 159 | 7.927 | 2 | 0.019 |
| Strategic | 159 | 2.391 | 2 | 0.303 |
| Surface | 159 | 16.853 | 2 | <0.001 |

a. The test statistic is adjusted for ties.

Table S3 – Dunn’s post-hoc pair-wise comparison for Deep and Surface scales across sites.

|  |  | χ^2^ | Std Error | Std Test Stat | Sig. | Adj. Sig.^a^ | r |
| --- | --- | --- | --- | --- | --- | --- | --- |
| Deep | Dublin – Bahrain | -19.571 | 7.804 | -2.508 | 0.012 | 0.036 | -0.198 |
|  | Dublin – Malta | -23.183 | 12.200 | -1.900 | 0.057 | 0.172 | -0.151 |
|  | Bahrain – Malta | -3.611 | 12.615 | -0.286 | 0.775 | 1.000 | -0.023 |
| Surface | Malta – Bahrain | 44.047 | 12.648 | 3.483 | <0.001 | 0.001 | 0.276 |
|  | Malta – Dublin | 50.049 | 12.232 | 4.092 | <0.001 | 0.000 | 0.324 |
|  | Bahrain – Dublin | 6.002 | 7.824 | 0.767 | 0.433 | 1.000 | 0.061 |

a. Significance values have been adjusted by the Bonferroni correction for multiple samples

Table S4 – Mann-Whitney U test for ASSIST scales across gender groups.

|  |  | Mann-Whitney | | |
| --- | --- | --- | --- | --- |
|  | *N* | Test statistic | Std test stat. | Sig. |
| Deep | 142 | 2056 | -1.476 | 0.14 |
| Strategic | 142 | 2349 | -0.247 | 0.805 |
| Surface | 142 | 1975 | -1.811 | 0.070 |

Table S5 – Kruskal-Wallis test for distribution differences across English Language CEFR levels.

|  |  | Kruskal-Wallis | | |
| --- | --- | --- | --- | --- |
|  | N | χ^2 a^ | df | Sig. |
| Deep | 142 | 7.556 | 2 | 0.023 |
| Strategic | 142 | 5.508 | 2 | 0.064 |
| Surface | 142 | 1.434 | 2 | 0.488 |

a. The test statistic is adjusted for ties.

Table S6 – Dunn’s post-hoc pair-wise comparison for Deep scale across English Language CEFR levels.

|  |  | χ^2^ | Std Error | Std Test Stat | Sig. | Adj. Sig.^a^ | r |
| --- | --- | --- | --- | --- | --- | --- | --- |
| Deep | C2-C1 | 11.939 | 8.463 | 1.411 | 0.158 | 0.475 | 0.118 |
|  | C2-B2 | 22.229 | 8.154 | 2.726 | 0.006 | 0.019 | 0.229 |
|  | C1-B2 | 10.289 | 9.118 | 1.128 | 0.259 | 0.777 | 0.095 |

a. Significance values have been adjusted by the Bonferroni correction for multiple samples

Table S7 – Kruskal-Wallis test for ASSIST scales distribution across Prior Education Background.

|  |  | Kruskal-Wallis | | |
| --- | --- | --- | --- | --- |
|  | N | χ^2 a^ | df | Sig. |
| Deep | 142 | 13.866 | 5 | 0.016 |
| Strategic | 142 | 2.558 | 5 | 0.768 |
| Surface | 142 | 4.186 | 5 | 0.523 |

a. The test statistic is adjusted for ties.

Table S8 – Dunn’s post-hoc pair-wise comparison for Deep scale across Prior Education Background.

|  | Sample 1 – Sample 2 | χ^2^ | Std Error | Std Test Stat | Sig. | Adj. Sig.^a^ | r |
| --- | --- | --- | --- | --- | --- | --- | --- |
| Deep | CHSD-IB | 23.106 | 12.36 | 1.869 | 0.062 | 0.923 | 0.157 |
|  | CHSD-AHSD | 26.662 | 10.973 | 2.430 | 0.015 | 0.227 | 0.204 |
|  | CHSD-Prior Foundation Programme | -33.781 | 10.174 | -3.321 | <.001 | 0.013 | -0.279 |
|  | CHSD-Other | -35.121 | 14.729 | -2.384 | 0.017 | 0.257 | -0.2 |
|  | CHSD-Tawjihiya | -38.635 | 13.911 | -2.777 | 0.005 | 0.082 | -0.233 |
|  | IB-AHSD | 3.555 | 12.007 | 0.296 | 0.767 | 1 | 0.248 |
|  | IB-Prior Foundation Programme | -10.675 | 11.281 | -0.946 | 0.344 | 1 | -0.079 |
|  | IB-Other | -12.014 | 15.515 | -0.774 | 0.439 | 1 | -0.064 |
|  | IB-Tawjihiya | -15.528 | 14.74 | -1.053 | 0.292 | 1 | -0.088 |
|  | AHSD-Prior Foundation Programme | -7.12 | 9.742 | -0.731 | 0.465 | 1 | -0.061 |
|  | AHSD-Other | -8.459 | 14.435 | -0.586 | 0.558 | 1 | -0.049 |
|  | AHSD-Tawjihiya | -11.973 | 13.598 | -0.881 | 0.379 | 1 | -0.074 |
|  | Prior Foundation Programme-Other | -1.339 | 13.837 | -0.097 | 0.923 | 1 | -0.008 |
|  | Prior Foundation Programme-Tawjihiya | -4.853 | 12.962 | -0.374 | 0.708 | 1 | -0.031 |
|  | Other-Tawjihiya | 3.514 | 16.777 | 0.209 | 0.834 | 1 | 0.018 |

a. Significance values have been adjusted by the Bonferroni correction for multiple samples

Table S9 – Mann-Whitney U test for ASSIST scales across Academic Achievement on Entry.

|  |  | Mann-Whitney | | |
| --- | --- | --- | --- | --- |
|  | *N* | Test statistic | Std test stat. | Sig. |
| Deep | 142 | 2206.5 | -0.113 | 0.910 |
| Strategic | 142 | 2425 | 0.837 | 0.403 |
| Surface | 142 | 2423.5 | 0.830 | 0.407 |

Table S10 – Normality test for Academic Performance

|  | Shapiro-Wilk | | |
| --- | --- | --- | --- |
|  | Statistic | Df | Sig. |
| Academic Performance | 0.984 | 142 | 0.090 |

Table S11 – Independent samples t-test with Welch’s correction for Academic Performance across Site

|  | Dublin  (n, M, SD) | Bahrain  (n, M, SD) | Mean diff (95% CI) | *t*(df) | *p* | Hedges’ *g* (95% CI) |
| --- | --- | --- | --- | --- | --- | --- |
| Academic Performance | 83, 66.0, 11.8 | 59, 64.4, 11.8 | 1.55 [−2.42, 5.53] | 0.772(125.46) | 0.441 | 0.13 [−0.20, 0.46] |

Table S12 – Independent samples t-test with Welch’s correction for Academic Performance across Gender

|  | Female  (n, M, SD) | Male  (n, M, SD) | Mean diff (95% CI) | *t*(df) | *p* | Hedges’ *g* (95% CI) |
| --- | --- | --- | --- | --- | --- | --- |
| Academic Performance | 86, 64.2, 12.4 | 56, 67.0, 10.7 | −2.81 [−6.68, 1.05] | -1.439(129.40) | 0.153 | −0.24 [−0.57, 0.10] |

Table S13 – Mean and standard deviation for Academic Performance across English Language CEFR level.

| Factor: Prior Education | n | M | SD |
| --- | --- | --- | --- |
| B2 | 43 | 63.2 | 13.01 |
| C1 | 38 | 67.9 | 10.00 |
| C2 | 61 | 65.2 | 11.81 |

Table S14 – One-way ANOVA test for Academic Performance across English Language CEFR level.

|  | Sum of Squares | df | Mean Square | F | Sig. |  |
| --- | --- | --- | --- | --- | --- | --- |
| Between Groups | 442.80 | 2 | 221.393 | 1.605 | 0.205 |  |
| Within Groups | | 17702.08 | 136 | 130.162 |  |  |
| Total | | 19613.44 | 141 |  |  |  |

Omnibus test: *F*(2,139) =1.605, *p* = 0.205, *η*^2^ = 0.023

Table S15 – Mean and standard deviation for Academic Performance across Prior Education Background

| Factor: Prior Education | n | M | SD |
| --- | --- | --- | --- |
| AHSD | 30 | 65.47 | 13.40 |
| IB | 19 | 60.32 | 10.42 |
| CHSD | 26 | 63.96 | 11.33 |
| Prior Foundation Programme | 43 | 64.23 | 10.59 |
| Tawjihiya | 13 | 74.15 | 11.73 |
| Other | 11 | 70.73 | 9.85 |

Table S16 – One-way ANOVA test for Academic Performance across Prior Education Background

|  | Sum of Squares | df | Mean Square | F | Sig. |
| --- | --- | --- | --- | --- | --- |
| Between Groups | 1911.362 | 5 | 382.272 | 2.937 | 0.015 |
| Within Groups | 17702.08 | 136 | 130.162 |  |  |
| Total | 19613.44 | 141 |  |  |  |

Omnibus test: *F*(5,136) =2.94, *p* = 0.015, *η*^2^ = 0.097

Table S17 – Games-Howell significant pairwise contrasts for Academic Performance across Prior Education Background

|  | Mean difference | *Adj. p* | Hedges’ *g* |
| --- | --- | --- | --- |
| Tawjihiya – IB | 13.84 | 0.024 | 1.23 |

Table S18 – Independent samples t-test with Welch’s correction for Academic Performance across Levels of Academic Achievement on Entry

|  | Low (n, M, SD) | High  (n, M, SD) | Mean diff (95% CI) | *t*(df) | *p* | Hedges’ *g* (95% CI) |
| --- | --- | --- | --- | --- | --- | --- |
| Academic Performance | 47, 59.3, 10.7 | 95, 68.3, 11.2 | 8.95 [5.10, 12.81] | 4.61(95.48) | <0.001 | 0.81 [0.44, 1.17] |

Table S19 – Pearson coefficients for Academic performance in Foundation Programme and ASSIST scales.

| Pearson Correlation |  |  | | |  | | |  | | |  | | |
| --- | --- | --- | --- | --- | --- | --- | --- | --- | --- | --- | --- | --- | --- |
|  |  | Academic  Performance | | Deep | | | Strategic | | | Surface | | |  |
| Academic Performance | Pearson Correlation | 1 | | 0.082 | | | .225^**^ | | | -0.158 | | |  |
|  | Sig. (2-tailed) | | 0.33 | | | 0.007 | | | 0.06 | | |  |  |
|  | N | 142 | | 142 | | | 142 | | | 142 | | |  |
| Deep | Pearson Correlation | 0.082 | | 1 | | | .208^*^ | | | 0.062 | | |  |
|  | Sig. (2-tailed) | 0.33 | |  | | | 0.013 | | | 0.46 | | |  |
|  | N | 142 | | 142 | | | 142 | | | 142 | | |  |
| Strategic | Pearson Correlation | .225^**^ | | .208^*^ | | | 1 | | | -.208^*^ | | |  |
|  | Sig. (2-tailed) | 0.007 | | 0.013 | | |  | | | 0.013 | | |  |
|  | N | 142 | | 142 | | | 142 | | | 142 | | |  |
| Surface | Pearson Correlation | -0.158 | | 0.062 | | | -.208^*^ | | | 1 | | |  |
|  | Sig. (2-tailed) | 0.06 | | 0.46 | | | 0.013 | | |  | | |  |
|  | N | 142 | | 142 | | | 142 | | | 142 | | |  |

**. Correlation is significant at the 0.01 level (2-tailed).

*. Correlation is significant at the 0.05 level (2-tailed).

Table S20 – Model Summary for multivariate linear regression for Academic Performance

| Model | R | R Square | Adjusted R Square | Std. Error of the Estimate |
| --- | --- | --- | --- | --- |
| 1 | .419^a^ | 0.176 | 0.145 | 10.9039 |
| 2 | .477^b^ | 0.228 | 0.182 | 10.67009 |

a. Predictors: (Constant), Academic Achievement on Entry, Prior Education Background, Gender, CEFR, Site

b. Predictors: (Constant), Academic Achievement on Entry, Prior Education Background, Gender, CEFR, Site, Strategic, Surface, Deep

Table S21 – Model 1 coefficients of multivariate linear regression for Academic Performance

| Predictor | *B* | *SE* | *β* | *t* | *p* |
| --- | --- | --- | --- | --- | --- |
| Intercept | 51.62 | 5.66 | — | 9.1 | <.001 |
| Site | 0.76 | 2.04 | 0.03 | 0.37 | .71 |
| Gender | 2.80 | 1.91 | 0.12 | 1.47 | .144 |
| CEFR | 0.87 | 1.15 | 0.06 | 0.76 | .449 |
| Prior Education Background | 1.51 | 0.62 | 0.20 | 2.44 | .016 |
| Academic Achievement on Entry | 8.04 | 2.09 | 0.32 | 3.86 | <.001 |

*R* = 0.419, *R*^2^ = 0.176, adj. *R*^2^ = 0.145, *F*(5,136) = 5.79, p < 0.001, *SE_est_* = 10.90

Table S22 – Model 2 coefficients of multivariate linear regression for Academic Performance

| z | *B* | *SE* | *β* | *t* | *p* |
| --- | --- | --- | --- | --- | --- |
| Intercept | 42.64 | 10.65 | — | 4.00 | <.001 |
| Site | 0.73 | 2.04 | 0.03 | 0.36 | 0.721 |
| Gender | 2.56 | 1.92 | 0.11 | 1.34 | 0.184 |
| CEFR | 1.00 | 1.15 | 0.07 | 0.85 | 0.396 |
| Prior Education Background | 1.26 | 0.61 | 0.16 | 2.06 | 0.042 |
| Academic Achievement on Entry | 8.05 | 2.05 | 0.32 | 3.93 | <.001 |
| Deep | 0.23 | 0.28 | 0.07 | 0.81 | 0.42 |
| Strategic | 0.43 | 0.22 | 0.16 | 1.95 | 0.053 |
| Surface | -0.29 | 0.21 | -0.12 | -1.41 | 0.162 |

*R* = 0.477, *R*^2^ = 0.228, adj. *R*^2^ = 0.182, *F*(8,133) = 4.91, p < 0.001, *SE_est_* = 10.67
